# Does the Anti‐Tau Strategy in Progressive Supranuclear Palsy Need to Be Reconsidered? No

**DOI:** 10.1002/mdc3.13326

**Published:** 2021-08-31

**Authors:** Günter U. Höglinger

**Affiliations:** ^1^ German Center for Neurodegenerative Diseases Munich Germany; ^2^ Department of Neurology Hannover Medical School Hanover Germany; ^3^ Center for Systems Neuroscience Hanover Germany

**Keywords:** progressive supranuclear palsy, microtubule‐associated protein tau, tau‐antibodies, tau‐antisense oligonucleotides, tau‐splice modifiers

Progressive supranuclear palsy (PSP) has been initially described as a neurodegenerative disease of unknown etiology, affecting the brain stem, basal ganglia, and cerebellum, leading to supranuclear gaze palsy, dysarthria, dysphagia, dystonic rigidity of the neck and upper trunk, postural instability, and a mild degree of dementia.^1^ Meanwhile, the clinical spectrum and neurodegenerative changes in PSP have been more precisely described.^2^, ^3^, ^4^ The neurobiological definition of PSP has emerged to focus a unique neuropathological pattern characterized by formation of insoluble aggregates composed mainly of 4‐repeat isoforms of the microtubule‐associated protein tau in the shape of globose neurofibrillary tangles, neuropil threads, oligodendroglial coiled bodies, and specifically tufted astrocytes.^4^, ^5^ The predominance of 4‐repeat tau aggregates led to the joint classification of a group of neurodegenerative diseases, including but not limited to PSP, corticobasal degeneration, argyrophilic grain disease, and globular glial tauopathies, as 4R‐tauopathies.^3^, ^5^ Because of their rapid clinical progression and the limited symptomatic therapeutic options available, there is a high unmet medical need to develop disease‐modifying therapies for 4R‐tauopathies.

The temporal and anatomical distribution of tau pathology in PSP appears to follow characteristic patterns and sequential stages,^4^ suggesting a propagation of tau pathology along axonal tracks. Because brain homogenates from patients with PSP can induce tau pathology in transgenic mice expressing wild‐type human tau,^6^ a prion‐like propagation of spreading‐competent tau species via the extracellular cerebral compartment has been assumed to constitute an important disease mechanism in PSP and other tauopathies.^4^, ^5^, ^6^

Therefore, 2 recent, sufficiently powered, randomized, controlled trials in PSP tested the hypothesis that passive immunization using monoclonal antibodies targeting the N‐terminus of tau would be efficacious to block the spreading of tau pathology and the associated disease progression. The antibody BIIB092 (gosuranemab), a humanized version of a murine antibody raised to target tau released from induced pluripotent stem cells from a familial patient with Alzheimer's disease,^7^ has been shown to strikingly reduce the concentration of free tau in the cerebrospinal fluid^8^ but failed to slow down the progression of clinical disease severity in a phase 2 trial.^9^ Also, the antibody ABBV‐8E12 (tilavonemab), a humanized version of a mouse monoclonal antibody raised against full‐length human tau,^10^ failed to show clinical efficacy in a phase 2 trial.^11^

Therefore, the concept to target tau to develop disease‐modifying therapies has been challenged. Although this series of unmet endpoints is painfully perceived as a drawback for the field, the results need to be put into the right perspective to avoid inordinate conclusions that risk to overshoot the mark, thereby preventing progress into the very right direction.

First, PSP is a primary 4‐repeat tauopathy from a neuropathological perspective.^4^, ^5^ There is no pathogen other than tau identified in PSP that would sufficiently explain neuronal dysfunction and death and subsequent neurological deficits. In a recent clinico‐pathological case series, we concluded after exhaustive characterization of potential alternative pathological features in the brains of PSP patients that copathology in PSP, albeit present not infrequently, does not sufficiently explain presence and progression of clinical symptoms in PSP.^12^ Primary dysfunction of tau, on the contrary, is well proven to be sufficient to explain progressive neuronal dysfunction and degeneration, as evidenced by mutations in the tau gene *MAPT* with an autosomal dominance trait pattern.^13^ Because the overwhelming majority of PSP cases are sporadic, mutations with loss‐of‐function or gain‐of‐function consequences for the protein product do not obviously explain the pathogenesis of PSP. Still, a large genome‐wide association study identified common variation at the *MAPT* locus to strongly increase the risk for PSP (odds ratio, 5.46).^14^ The strongest signal of this association points to an inversion polymorphism in the *MAPT* region, which appears to affect the expression of tau and its isoform balance.^14^ An epigenome‐wide DNA‐methylation study did not identify significantly different methylation at *MAPT* in PSP versus controls, but methylation changes in pathways that indirectly appear to affect tau expression.^15^ An epidemiological association of an unexpectedly high prevalence of a PSP‐like tauopathy in Guadeloupe with the consumption of fruits containing the mitochondrial complex I inhibitor annonacin pointed to possible environmental mechanisms explaining disease risk.^16^ Importantly, annonacin induced a shift of tau and mitochondria from axons to somata and an increase in 4‐repeat tau isoforms in cultured neurons, thereby reproduction hallmarks of 4‐repeat tauopathies.^17^, ^18^ In sum, all lines of evidence available so far point to a dysfunction of tau as an essential, if not primary, deficit leading to neuronal dysfunction, neurodegeneration, and neurological deficits in PSP.

Therefore, it appears not appropriate to question tau as the prime target for the development of therapeutic interventions, just because the first 2 trials did not hit the right spot within the protein. We may conclude with due care that antibodies targeting N‐terminal tau in the currently applied paradigm do not modify disease progression in established PSP. Although patients and scientists would have hoped very much for success at the first try, these results provide important insights to move the field forward.

Recent years have provided insights to suggest that the N‐terminus of tau might not be the appropriate target, both with regard to the abundance in the extracellular space and the biological relevance in the pathological seeding and aggregation mechanisms of tau.^19^, ^20^, ^21^ On the contrary, there is emerging preclinical evidence to suggest that antibodies targeting the mid‐region of tau next to the aggregation‐prone repeat domain might possess superior properties to block tau spreading.^22^, ^23^ A corresponding humanized antibody (UCB0107, bepranemab) has recently entered the clinical evaluation in a phase 1 trial in PSP (NCT04185415, NCT04658199). Also silencing of tau expression by intrathecal injection of antisense oligonucleotides, proven efficacious in tauopathy mouse models,^24^ is currently being evaluated in a phase 1 study in PSP (NCT04539041). In the preclinical setting, there are upcoming tau‐targeting opportunities by stimulating cellular defense mechanisms against misfolded tau,^25^ by reducing tau expression,^26^ or by altering tau splicing and thereby isoform expression.^27^ A more comprehensive presentation of tau‐targeting opportunities is presented elsewhere.^5^

Of course, our perspective has to remain wide open so as not to miss any relevant therapeutic opportunities, including the relevance of tau loss‐of‐function mechanisms,^28^ cell types other than neurons (eg, astrocytes),^29^, ^30^ neuroinflammation,^31^ and energy failure.^17^, ^32^ Furthermore, it appears possible that the current therapeutic interventions have just been initiated at a too advanced stage of the disease, when extensive cell‐to‐cell transmission of the pathologic tau is already too established by the time the diagnosis is made currently, arguing for further research into early diagnosis.^33^

In conclusion, on our way into unknown territories, failed clinical trials need to be accepted as important learning opportunities to find the right path to reach the target safely and efficaciously. Failed trials need to stimulate the community to reconsider the approach toward the target, not the well‐set target itself, without having tried all opportunities in an appropriate manner. Patients rightfully request this effort from research.

## Disclosures

### Ethical Compliance Statement

The author confirms that patient consent was not required for this work. The author confirms that the approval of an institutional review board was not required for this work. I confirm that I have read the Journal's position on issues involved in ethical publication and affirm that this work is consistent with those guidelines.

### Funding Sources and Conflicts of Interest

This work was supported by the the German Federal Ministry of Education and Research (Bundesministerium für Bildung und Forschung, BMBF, 01EK1605A HitTau), the VolkswagenStiftung/Lower Saxony Ministry for Science (Niedersächsisches Vorab), the Petermax‐Müller Foundation (Etiology and Therapy of Synucleinopathies and Tauopathies). G.U.H. has served as consultant for Abbvie, Alzprotect, Asceneuron, Bial, Biogen, Biohaven, Kyowa Kirin, Lundbeck, Novartis, Retrotope, Roche, Sanofi, and UCB; has received honoraria for scientific presentations from Abbvie, Bayer Vital, Bial, Biogen, Bristol Myers Squibb, Kyowa Kirin, Roche, Teva, UCB, and Zambon; and has participated in industry‐sponsored research projects from Abbvie, Biogen, Biohaven, Novartis, Roche, Sanofi, and UCB.

### Financial Disclosures for the Previous 12 Months

G.U.H. has received institutional support from the German Center for Neurodegenerative Diseases and Hannover Medical School; has served as consultant for Abbvie, Alzprotect, Asceneuron, Bial, Biogen, Biohaven, Kyowa Kirin, Lundbeck, Novartis, Retrotope, Roche, Sanofi, and UCB; has received honoraria for scientific presentations from Abbvie, Bayer Vital, Bial, Biogen, Bristol Myers Squibb, Kyowa Kirin, Roche, Teva, UCB, and Zambon; has participated in industry‐sponsored research projects from Abbvie, Biogen, Biohaven, Novartis, Roche, Sanofi, and UCB; has received publication royalties from Academic Press, Kohlhammer, and Thieme; holds a patent (G.U. Höglinger, M. Höllerhage, T. Rösler, “Treatment of synucleinopathies,” US Patent No. US 10,918,628 B2, date of patent: February 16, 2021); has received research support from Deutsche Forschungsgemeinschaft (German Research Foundation) under Germany's Excellence Strategy within the framework of the Munich Cluster for Systems Neurology (EXC 2145 SyNergy–ID 390857198) and within the Hannover Cluster RESIST (EXC 2155–ID 390874280); Deutsche Forschungsgemeinschaft (HO2402/18–1 MSAomics); the German Federal Ministry of Education and Research (BMBF, 01KU1403A EpiPD; 01EK1605A HitTau; 01DH18025 TauTherapy); the EU/EFPIA/Innovative Medicines Initiative Joint Undertaking (IMPRIND grant no. 116060); CurePSP (DNA‐methylation in PSP brains, miRNAs in PSP brains); Niedersächsisches Ministerium für Wissenschaft und Kunst: REBIRTH–Forschungszentrum für translationale regenerative Medizin; VolkswagenStiftung (Niedersächsisches Vorab); Petermax‐Müller Foundation (Etiology and Therapy of Synucleinopathies and Tauopathies); the German Parkinson Society; ProAPS; the German PSP Association (PSP Gesellschaft); ProPSP; the ParkinsonFonds Germany (hypothesis‐free compound screen, alpha‐synuclein fragments in Parkinson's disease).
